# The comparison of dispersal rate between invasive and native species varied by plant life form and functional traits

**DOI:** 10.1186/s40462-023-00424-y

**Published:** 2023-11-03

**Authors:** Bo Zhang, Alan Hastings, Edwin D. Grosholz, Lu Zhai

**Affiliations:** 1 Department of Environmental Science and Policy, University of California, Davis, CA USA; 2 Department of Integrative Biology, Oklahoma State University, Stillwater, OK USA; 3 Santa Fe Institute, Santa Fe, NM USA; 4 Department of Natural Resource Ecology and Management, Oklahoma State University, Stillwater, OK USA

**Keywords:** Dispersal rate, Species invasion, Plant height, Seed length, Plant life form, Leaf dry matter content, Longevity

## Abstract

A long dispersal distance is widely used to indicate high invasiveness, but it ignores the temporal dimensions of plant invasion. Faster dispersal rates (= distance/time) of invasive species than native ones have been widely used in modeling species invasion and planning control management. However, the comparison of dispersal rate between invasive and native plants, particularly for dispersal on a local or landscape scale, has not been tested with a comprehensive dataset. Moreover, both the effects of plant functional traits on the dispersal rate and variation in the functional-trait effects between invasive and native plants remain elusive. Compiling studies from 30 countries globally, we compared seed dispersal rates (km/year) on a local or landscape scale between 64 observations of invasive and 78 observations of native plants given effects of plant life forms, disturbance levels, and measurement methods. Furthermore, we compared the effects of functional traits on dispersal rate between invasive and native species. We found that: (1) Trait values were similar between the invasive and native plants except for the greater height of woody native plants than woody invasive ones; (2) Compared within the same plant life form, the faster dispersal rates of invasive species were found in herbaceous plants, not in woody plants, and disturbance level and measurement methods did not affect the rate comparison; (3) Plant height and seed length had significant effects on dispersal rates of both invasive and native plants, but the effect of leaf dry matter content (LDMC) was only significant on herbaceous invasive plants. The comparison of dispersal rate between invasive and native plants varied by plant life form. The convergent values but divergent dispersal effects of plant traits between invasive and native species suggest that the trait effects on invasiveness could be better understood by trait association with key factors in invasiveness, e.g., dispersal rate, than the direct trait comparison between invasive and native plants.

**Supplementary Information:**

The online version contains supplementary material available at 10.1186/s40462-023-00424-y.

## Introduction

Understanding mechanisms of species invasions is of fundamental interest to biological conservation [48], and the understanding can be improved by a comprehensive comparison between invasive and native species, e.g., van Kleunen et al. [64], Davidson et al. [10], Broadbent et al. [5]. For example, previous studies revealed that, compared with native plants, invasive ones had significantly higher values of performance-related traits, including seedling relative growth rate [23], resource use efficiency [27], and seed production per seed mass [37]. Moreover, the comparisons suggest that a strong dispersal ability has been recognized as a fundamental driver of biological invasion [4, 53], due to dispersal impacts on species range expansions [30, 34]. Previous studies focused more on dispersal distance, for example, Nunez‐Mir et al. [47] showed that invasive species had longer dispersal distance than native ones. However, dispersal distance ignores the temporal dimension of dispersal, which can significantly affect the dispersal ability conferring invasiveness, i.e., if a species disperses the same distance as another species, but in a shorter time, it has a better dispersal ability [51]. Therefore, by considering the temporal dimension, dispersal rate (= distance/time) could improve the understanding of invasion mechanisms and be another component of a unified framework for biological invasions [2]. However, to our knowledge, no studies have comprehensively compared dispersal rates between invasive and native plants across a broad range of species since dispersal is one of the most challenging ecological processes to measure in the field [8].

Dispersal rate information is critical to understand species distribution, abundance, and population dynamics [11, 18, 61]. For example, an intermediate dispersal rate can significantly increase total population abundance in heterogeneous environments than both low and high dispersal rates [73], and the difference of dispersal rate between two competing species could alter their coexistence trajectory [25]. Therefore, dispersal rate, which appears as “diffusion rate” in reaction–diffusion models, is one of the most widely used variables to project invasion dynamics in many mathematical models [28, 45, 72]. For instance, to model spread of an invader, a dispersal rate is one of the most important parameters in “Fisher’s equation” [20], and dispersal rate-based models are more successful in predicting population spread than life-history-based models [26]. A failure to incorporate dispersal rate into models of vegetation dynamics greatly compromises their predictive capability, leading to substantial modeling uncertainty [40]. In mathematical models of species invasion, invasive species have been widely parameterized with faster dispersal rates than native ones, given that invasive species are expanding to new habitats, but native ones tend to be stable in their habitats [21]. However, the variation in the comparison of dispersal rate between invasive and native species are largely unknown, and the variation may be driven by plant life forms (herbaceous vs. woody plants), disturbance levels (low vs. high disturbance levels), and measurement methods. Without considering these factors, we may get a biased comparison of dispersal rate [52]. Thus, our study compiled dispersal rate data from previous studies and compared dispersal rate between invasive and native plants, given the potential effects of above-mentioned factors.

Seed dispersal could be related to plant functional traits, including seed size and mass, plant longevity, plant height, and leaf traits. For example, seed mass and plant height are significantly related to maximum dispersal distances of multiple species [60]. Leaf traits, such as greater leaf dry matter content (LDMC) could reduce leaf N and water content, and further decrease plant palatability [15]. As a result, leaves with lower LDMC are preferred by herbivores, ultimately leading to increased dispersal rates. Thus, these traits are also expected to relate to dispersal rate. Compared with small seeds, large seeds are preferentially selected by animal dispersers [54], potentially contributing to their more rapid dispersal [57]. Given similar seed size, seed mass is often negatively correlated with dispersal rate, since wind-dispersed seeds tend to spread faster with lighter mass [66]. The above-mentioned expectation is well-supported in a broad range of ecosystem types [57], but exceptions occur, e.g., Wyse et al. [70]. Plant longevity shows a negative correlation with the dispersal rate because short-lived plants are likely to have better dispersal capacity [17]. For leaf traits, higher leaf dry matter contents (LDMC) suggest relatively low nutrient contents that tend to reduce plant palatability and attractiveness to herbivores, leading to lower seed dispersal [67]. Therefore, these functional traits could be used to predict dynamics of seed dispersal [1]. More importantly, the potentially divergent values of these dispersal-related traits (i.e., functional dissimilarity) between invasive and native plants may explain their hypothesized differences in dispersal rate. The comparison of these traits between invasive and native plants is expected to contribute to explaining and predicting species invasion from dispersal perspective [53].

We assembled dispersal rates (km/year) of invasive and native species from published data across a broad range of plant species at a global scale. The species invasiveness (invasive vs. native) was based on specific location based on the data source. Furthermore, we extracted data of different plant functional traits for the species studied from public trait databases. Capitalizing on the data of dispersal rate and plant functional traits of invasive and native plants, we tested two hypotheses: (1) Invasive plants have faster dispersal rates than native ones; (2) Functional traits are different between invasive and native plants and affect dispersal rate.

## Methods

### Dispersal rate data

To identify dispersal rate differences between invasive and native plants, we searched research articles written in English in Google Scholar database from 1980 to 2020 and used the keyword combination: (“invasive”, “exotic”, “non-native”, “alien”, “foreign”, “non-indigenous”) AND (“native”, “indigenous”, “native”, “non-exotic”, “non-alien”, “non-foreign”) AND (“dispersal rate”, “slow dispersal”, “fast dispersal”) AND (“plant”, “grass”, “herb”, “tree”, “shrub”, “forest”) in sections of title, abstract, and keyword. It is important to note that very few references (three found in our search) calculated dispersal rates of both invasive and native plants in similar study areas. Therefore, we conducted searches for dispersal rates of invasive and native plants, independently, and this searching approach has already been used by other studies to compile functional trait data of invasive and native species (e.g., Nunez‐Mir et al. [47]). Note that we followed standard protocol for conducting systematic reviews [41] to only include species that are clearly defined as an invasive species in the study location, and exclude species that could be defined as invasive due to their high dispersal rate. Additionally, it is important to be aware that some native species may be invasive elsewhere outside of their native range, potentially confounding results. Based on the searching outputs, we went through the sections of Abstract, Methods, and Results of each article. Finally, we compiled data of 64 observations for invasive plants (39 species, 67% herbaceous and 33% woody), and 78 observations for native plants (74 species, 74% herbaceous and 36% woody), derived from 35 published studies for invasive plants and 10 published studies for native plants (Reference list is included in Additional file 1). The recognition of invasive and native species was determined by species descriptions from these studies, i.e., invasive species are those not native and causing threats to local ecosystems, native species are those that have always been parts of the ecosystems where studies were conducted. These published works are from 30 countries covering six continents (except Antarctica).

Given that disturbance is a critical factor in plant dispersal, we classified habitats where dispersal rate was collected in each study into two groups (low *vs.* high disturbed group). The habitats that are close to areas with high human activities (e.g., farm, dock, wasteland, etc.) or characterized by highly dynamic environments (e.g., coastal dune, pasture, and river) are classified into the high disturbed group. Alternatively, the habitats that are generally under relatively stable environments, such as forest, shrubland, and grassland, are classified into the low disturbed group. The disturbance group was included in the data analysis to consider its effect on the comparison of dispersal rate between invasive and native plants.

Additionally, we hypothesized that the difference in dispersal rate between invasive and native plants could be associated with the measuring protocol. Hence, we documented the protocol of how the dispersal rate was measured for all the available studies. We classified these protocols into two groups: Ground based and non-ground-based data. Some studies used field data (pollen or other ground-based measures) to measure dispersal rate as dividing the distance between the location where a species was recorded on the earliest date and the most distant point where a species was recorded by the residence time [9, 29], and measures from these studies are classified as ground-based data. Alternatively, studies that used aerial photographs or microsatellite information were defined as the nonground-based data group. For instance, some studies used a series of aerial photographs and population dynamics data at a fine scale to calculate an invaded area, then dispersal rate was calculated via dividing the square root of the invaded area by residence time [43, 50], other studies used microsatellite genotypes of seed arrays to determine the dispersal rate [13]. To keep the dispersal rate in a consistent and standard unit, we used “km/year” as the unit for all the collected data.

### Plant functional trait data

We compiled five functional traits that are relevant to dispersal rate from the Botanical Information and Ecology Network (BIEN) database [36]. The five traits are whole-plant height (m), leaf dry matter content (LDMC) (mg g^−1^), plant longevity (year), seed mass (mg), and seed length (mm). For each of these traits, we selected values from the same ecoregion with a study area. If not available, we used all observations from the trait database. Then, we calculated mean of the selected values for one species. In addition, we classified the plants studied into two growth forms: woody and herbaceous plants.

### Statistical analysis

#### Comparison of dispersal rate and functional traits between invasive and non- invasive species

The comparisons were made by linear mixed-effect (LME) models with a phylogenetic relatedness between the species studied, and a phylogenetic tree of these species is shown in the Fig. S1 of the Additional file 2. Both the dispersal rate and functional traits were used at the natural logarithmic scale, and species was used as a random factor in the analysis. Both two models (with a phylogenetic relatedness between the species studied and a phylogenetic tree of these species) used the plant groups (i.e., invasive *vs.* native plant) as fixed factors. Fixed factors of the dispersal rate model also consisted of plant life forms (i.e., herbaceous *vs.* woody species), disturbance groups (i.e., low *vs.* high disturbance), and measurement methods (i.e., ground vs. non-ground data). The homogeneity of residual variance was examined using the residual plots along with fitted values and fixed predictors of the models. The violations of normality were examined using the normal quantile plots. The above statistical analyses were implemented by the R program (R Core Team 2020), and the models were built by the ‘phyr’ package [31], and the figures were made by the ‘ggplot2’ package [68]. All analyses were considered significant at *p* < 0.05.

#### Structural equation models

A structural equation model (SEM) was built to compare the direct and indirect effects of the five functional traits on the dispersal rate. The dispersal effects of functional traits were compared between invasive and native species using a multigroup analysis. Correlation paths and their directions were hypothesized from previous studies, including from tree height to dispersal rate [6, 38, 59], LDMC to dispersal rate [67], seed length and seed mass to dispersal rate [12, 56, 66, 69]. The partial residuals of dispersal rate from the above LME model were used as standardized dispersal rates to mitigate the confounding effects studied above to focus on the effects of plant traits. The plant height, longevity, seed mass, and seed length were used at the natural logarithmic scale to satisfy the assumptions of normality and linearity. The SEM analysis and multigroup analysis were carried out through the piecewiseSEM package in R [33]. All analyses were considered significant at *p* < 0.05.

## Results

### The comparison of standardized dispersal rate

**Fig. 1:**
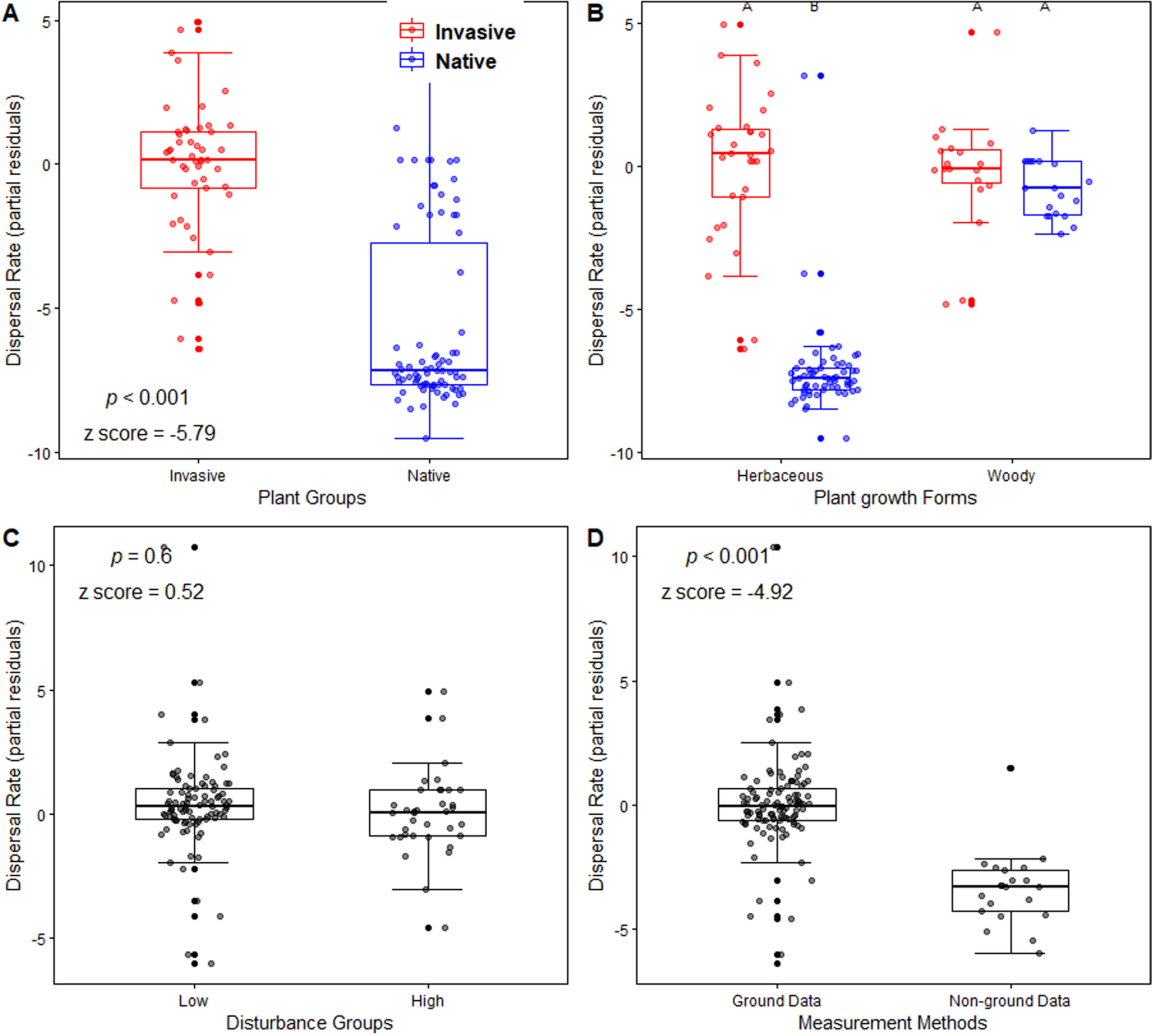
Partial residual plots showing the dispersal rate comparison by plant groups (**A**), plant life forms (**B**), disturbance groups (**C**), and measurement methods (**D**). Boxplots denote the median (centerline) and interquartile range (box), with upper and bottom whiskers (or error bars) extending to 1.5 × interquartile range measured out from upper and bottom sides of the box, respectively. Statistical significances of the comparisons were denoted by the *p* values calculated by the linear mixed model. The different letters indicate significant differences (*p* < 0.05)

Our results showed that invasive plants had a significantly faster dispersal rate than native ones (*p* < 0.001, z score = − 5.79, Fig. 1A). However, the comparison of the dispersal rate between the two plant groups varied with plant life forms, i.e., compared with native plants, faster dispersal rates of invasive ones were only observed in herbaceous species (*p* < 0.001, z score = 4.98), and not in woody species (Fig. 1B). Moreover, the comparison of dispersal rate between the two plant groups did not vary with disturbance and measurement methods. There were no significant rate differences between the two levels of disturbance (*p* = 0.6, z score = 0.52, Fig. 1C), but ground-based measures showed higher rates than non-ground measures (*p* < 0.001, z score = − 4.92, Fig. 1D). Summary of Z score and *p* value of predictors in the linear mixed-effect model are recoded in Table S2 in Additional file 2.

### The associations between dispersal rate and functional traits

**Fig. 2:**
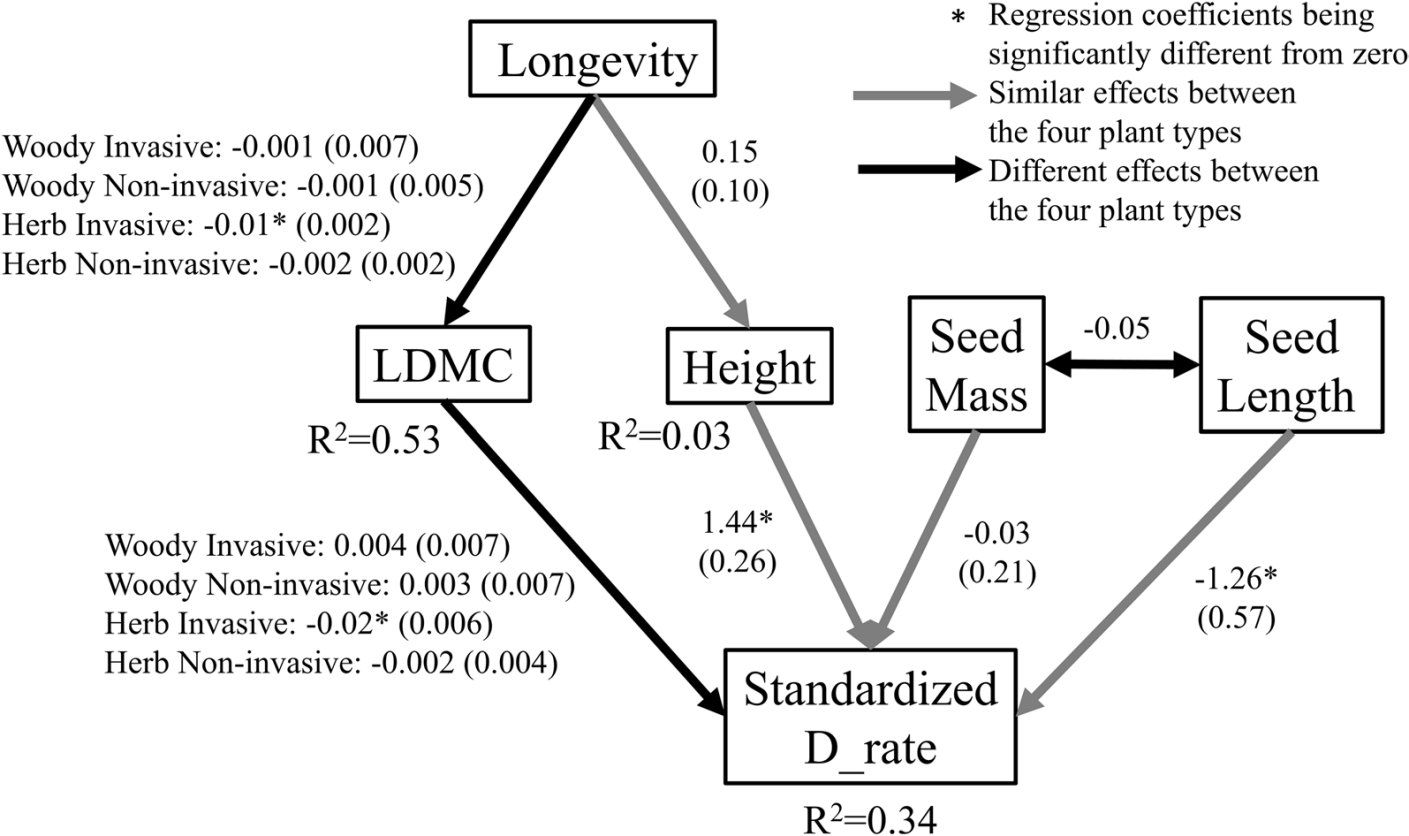
Structural Equation Model assesses the direct and indirect effects of functional traits on standardized dispersal rate (standardized D_rate) among the four plant types: woody invasive, woody native, herbaceous invasive, and herbaceous native plants. The solid lines denote similar effects, but the dashed lines denote significantly different effects among the four plant types. The values on the solid and dashed lines are regression coefficients (or slopes), and the values in the parentheses are standard errors of the regression coefficients. Height: whole plant height (m); LDMC: leaf dry mass per leaf fresh mass (mg g^−1^); Seed length (mm); Seed mass (mg); Plant longevity (year)

The structural equation model (SEM) explained 34% variation in the dispersal rate and goodness of fit of the SEM was validated (*p* = 0.39, Fisher's C = 12.74, df = 12). Based on the SEM, there are two pathways that were similar between invasive and native plants and had significant correlations with dispersal rate (solid lines in Fig. 2): (1) seed length was negatively related to dispersal rate (regression coefficient = − 1.26, *p* = 0.03, t-statistic = − 2.23, df = 81); (2) plant height was positively related to dispersal rate (regression coefficient = 1.44, *p* < 0.001, t-statistic = 5.65, df = 81). Note that compared with the non-significant factors, standard errors of the significant ones are smaller relative to their coefficient values, i.e., the ratios between standard error and coefficient value are smaller for significant factors than non-significant ones.

Notably, we detected pathways from plant functional traits to dispersal rate that were divergent among the four plant types defined by combinations of plant group (invasive *vs.* native plants) and growth form (woody *vs.* herbaceous plants) (dash lines in Fig. 2). Compared with the other three plant types, herbaceous invasive plants characterized a significant compound path from longevity to LDMC (regression coefficient = − 0.01, *p* < 0.001, t-statistic = − 4.79, df = 24), and then to dispersal rate (regression coefficient = − 0.02, *p* = 0.003, t-statistic = − 3.40, df = 21). Based on product of these two negative coefficients, the compound path suggests positive effect of longevity on dispersal rate.

### The comparisons of the functional traits

**Fig. 3:**
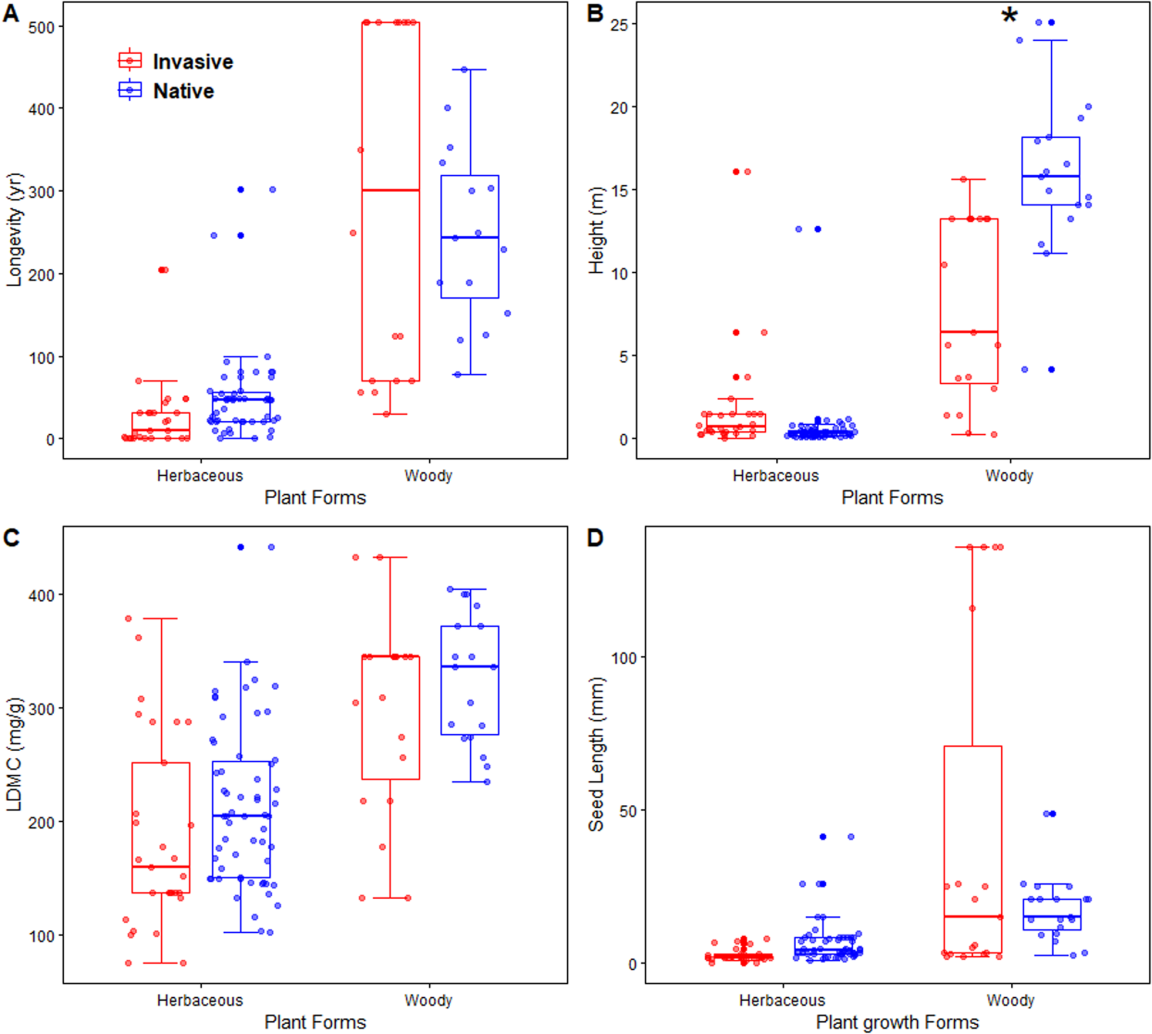
Comparison of **A** Longevity (year), **B** Plant height (m); **C** LDMC: leaf dry mass per leaf fresh mass (mg/g) and **D** Seed length (mm) among the four plant types. All other variables are as previously defined. The asterisk sign indicates the significant difference between invasive and native plants within a plant life form

From the above-mentioned trait and dispersal rate correlation analysis, there are four traits showing significant associations with dispersal rates, including longevity, height, LDMC, and seed length. Here we compared values of these functional traits between the four plant types: woody invasive, woody native, herbaceous invasive, and herbaceous native plants. Within each plant growth form, invasive and native plants show similar trait values except for plant height (Fig. 3, see *p* values and z scores of the comparisons in Table S1 in Additional file 2). For woody species, native plants had greater height than invasive ones (*p *< 0.001, z score = 6.830, Fig. 3B).

## Discussion

### Compared within a plant growth form, the faster dispersal rates of invasive species than native ones were found in herbaceous plants, not in woody plants

Our analysis showed that the dispersal rates of invasive plants were generally faster than that of native ones. This result parallels previous studies showing longer dispersal distances of invasive plants than native ones, e.g., Nunez‐Mir et al. [47]. Therefore, both faster rates and longer distances of seed dispersal facilitate plant invasion over a wider spatial range in a shorter time [58]. Compared with native plants, the faster dispersal rates of invasive plants further support the classic theory that successful invasive species would have greater dispersal ability to occupy vacant niches [16]. Notably, compared within a plant life form, the faster rates of invasive species were only found in herbaceous plants, not in woody plants. This result is consistent with that compared with herbaceous plants, woody ones are often poor dispersers, and woody invasion tend to be more related to changes in extrinsic conditions such as climate, fire, grazing, and long-distance dispersal mediated by human than local dispersal studied here [14, 55]. Therefore, the difference of dispersal rate between invasive and native woody plants may not be as large as previously expected, suggesting the important role of plant growth form in parameterizing dispersal rates of species invasion in mathematical models. In addition to dispersal rate, maximum dispersal is also significantly related to plant growth form [60]. Moreover, land disturbance and measurement methods did not affect the rate comparison, but we showed that there were faster rates calculated by ground-based than non-ground-based data (e.g., aerial image measurements). The method-caused difference may result from that small individuals (e.g., seedlings) are difficult to be identified with aerial images or other non-ground-based methods, resulting in underestimates of dispersal rate using non-ground measures. This limit may be overcome by recent advance in remote sensing technology, e.g., application of hyperspectral data to map invasive plants [22].

### The dispersal rates showed divergent and convergent associations with functional traits between invasive and native plants

The SEM analysis revealed both divergent and convergent trait effects on dispersal rates between the two plant groups. The effect of LDMC was divergent between the two plant groups. We found that the pathway from longevity to LDMC to wind dispersal rate was only significant on the invasive plants, but not on native ones. The negative association between LDMC and dispersal rate could result from that greater LDMC could reduce leaf N and water content, and further decrease plant palatability [15]. Therefore, relatively high LDMC is one of the critical anti-herbivory leaf traits [32, 49]. As a result, leaves with lower LDMC are preferred by herbivores, ultimately leading to increased dispersal rates. Notably, we showed that the LDMC effect was only significant in invasive plants. It may be caused by that compared with native plants, invasive ones may have less herbivory pressure in the early stage of invasion due to few specialized herbivores in their new ranges, i.e., the Enemy Release Hypothesis [71]. Consequently, invasive plants may evolve to allocate resources to growth or reproduction instead of defenses against enemies [44]. This allocation strategy results in declined LDMC and higher seed production, rendering faster dispersal rates with more attractiveness to generalist herbivores as the invasion is progressing [24]. Therefore, with the allocation strategy, the significant effect of LDMC may contribute to the faster dispersal rates of invasive plants than native ones. However, the dispersal-rate effects of plant height and seed length were convergent between invasive and native plants. Our study showed the positive effect of plant height on dispersal rate, particularly for wind-based dispersal, which is consistent with another cross-species study [62]. Meanwhile, mixed height effects on dispersal distance were reported (i.e., positive, negative, and neutral effect) in previous studies reviewed by Schupp et al. [57]. Shorter species could achieve fast dispersal rate through animals by attaching to their fur when seeds are small, leading to high invasion capability [42].

### The functional traits had convergent values between the invasive and native plants

The values of most function traits (longevity, LDMC, and seed mass), with the significant associations with dispersal rate, were similar between invasive and native plants grouped by plant growth form. The functional similarity is consistent with the results of Nunez‐Mir et al. [47], showing that these functional traits make minor contributions to the separation of invasive and native plants in a trait space. Therefore, the high functional similarity between invasive and native plants supports the ‘join-the-locals’ hypothesis, i.e., invasive plants share traits with native ones to adapt to local environments [35]. Note that the trait comparison between invasive and native species vary with specific traits studied, e.g., another pair-wise comparison-based study showed invasive plants have higher values for traits related to performance than native ones [64].

The invasive and native plants showed convergent values of LDMC but different effects of LDMC on dispersal rate as discussed above, i.e., the negative effects of LDMC on dispersal rate was only significant on invasive plants. Thus, compared with the trait values, the divergent responses to LDMC could be more important to understand the different dispersal rates between invasive and native plants. The specific responses to critical functional trait could inform the prediction of dispersal rate and the related species invasion. Therefore, distinguishing invasive and native species could rely on traits associations with key factors determining invasiveness, e.g., dispersal rate, rather than the direct comparison of traits between invasive and native species [7].

### Conclusions and implications

Taken together, the faster dispersal rates of invasive plants were only found in herbaceous plants, suggesting the comparison of dispersal rate between invasive and native plants varied by plant life form. Moreover, the convergent values but divergent dispersal effects of plant traits between invasive and native species suggest the trait effects on invasiveness could be understood by trait association with key factors in invasiveness, e.g., dispersal rate, rather than the direct trait comparison between invasive and native plants. Our study has important implications for modeling species invasion and invasion control. Our database can be used to calibrate mathematical models of species invasion [46] and develop a dispersal component in modeling large-scale vegetation dynamics given this component is one of the most undeveloped demographic processes, e.g., in Earth System Models (ESM) [39]. Notably, the significant associations between dispersal rate and functional traits in our study reveal the potentials of using easily-measured functional traits to predict hardly-measured dispersal rates. Moreover, the dispersal rate database compiled by our study can inform practices of invasion control. For example, field applications of biocontrol agents can use our dataset to determine an appropriate releasing speed of biocontrol agents to catch up with the spreads of invasive species [19].

When collecting data from previous studies, we noticed some critical issues about dispersal studies. First, there is a lack of studies investigating dispersal of a species in both its native range and new environment where it is invading. For example, our dataset only has one species found in both invasive and native plants. This study gap limits our ability to examine whether an invasive species also maintain high dispersal rates when they are native. Second, there is a need to report specific dispersal agent in dispersal studies because dispersal agent is a critical dispersal factor, potentially confounding other factors on dispersal [65]. However, the agent information were not available from most studies included in our dataset. Third, our dataset focuses on dispersal at a local or landscape scale (or short-distance dispersal events), and the short-distance dispersal is an important step of establishing invasion after a species arrives at a specific location. However, there is a limited data availability of long-distance dispersal events, describing the dispersal process before the species arrival. The long-distance dispersal of invasive species is increasingly dominated by human-mediated dispersal at a global scale [63]. The long-distance dispersal may still be informed by the trait-based analysis of the short-distance dispersal in our study. For example, given the climate change impacts, plant functional traits related to drought and heat stress could indicate plant preference during the global horticultural trade which is causing another wave of plant invasions [3]. Meanwhile, the human-mediated dispersal is affected by a wide range of social, economic, cultural, and environmental factors, which could be analyzed by future studies with the increasing data availability.

### Supplementary Information

**Additional file 1. **Original data of species dispersal rates and functional traits.

**Additional file 2: Fig. S1. **The phylogenetic tree of both the invasive and native species studied. **Table S1. **The *p* values and z scores of the trait comparisons between the invasive and non-invasive species within one plant forms, i.e., herbaceous and woody plants. **Table S2.** Summary of Z score and *p* value of predictors in the linear mixed-effect model.

## Data Availability

Data are provided as private-for-peer review. The material is currently stored at Dryad (https://datadryad.org/stash/share/WP2pW4QA0ADxIIers-CZaIaifoIs4j_ySHA8U_VXk-Y), and data will be permanently archived by Dryad if the paper is accepted for publication.
