# Supplementary material for: The comparison of dispersal rate between invasive and native species varied by plant life form and functional traits
Source: Mov Ecol. 2023 Nov 3;11:73. doi: 10.1186/s40462-023-00424-y (PMC10623791; doi:10.1186/s40462-023-00424-y)
Supplement: Supplementary file 2 — Additional file 2: Fig. S1. The phylogenetic tree of both the invasive and native species studied. Table S1. The p values and z scores of the trait comparisons between the invasive and non-invasive species within one plant forms, i.e., herbaceous and woody plants. Table S2. Summary of Z score and p value of predictors in the linear mixed-effect model. [file 40462_2023_424_MOESM2_ESM.docx]

**Appendix**


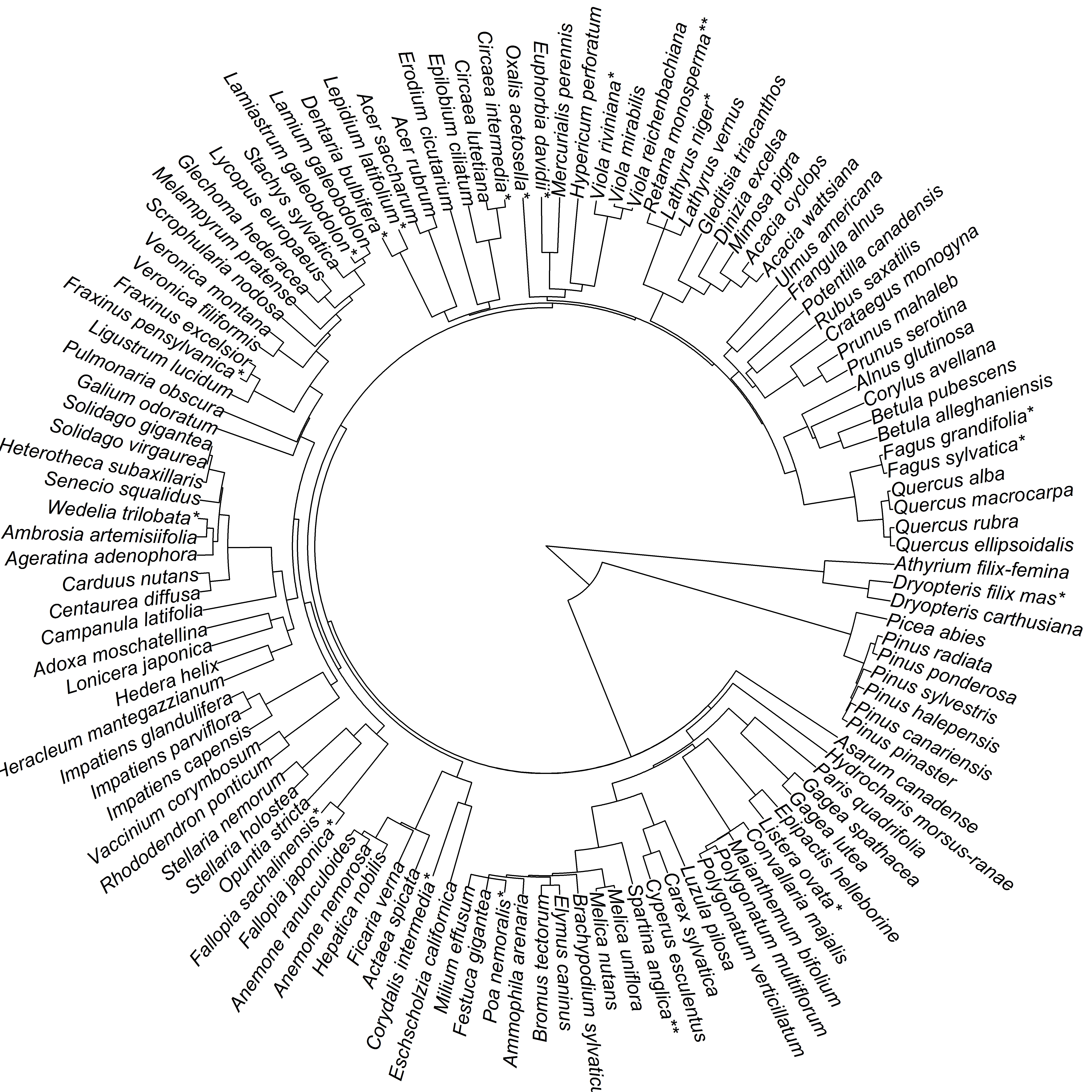


Figure S1: The phylogenetic tree of both the invasive and native species studied.

Table S1: The *p* values and z scores of the trait comparisons between the invasive and non-invasive species within one plant forms, i.e., herbaceous and woody plants.

| Plant functional Traits | Herbaceous Plants | | Woody Plants | |
| --- | --- | --- | --- | --- |
|  | p value | Z score | p value | Z score |
| Longevity | 0.551 | -0.597 | 0.995 | 0.006 |
| Height | 0.353 | -0.930 | **<0.001** | 6.830 |
| LDMC | 0.750 | -0.319 | 0.970 | 0.038 |
| Seed Length | 0.630 | -0.482 | 0.981 | 0.024 |

Table S2: Summary of Z score and p value of predictors in the linear mixed-effect model.

| Predictors | Z Scores | *p* Values |
| --- | --- | --- |
| Plant Group | -5.7846 | <0.0001 |
| Plant Growth Form | -0.3104 | 0.7562 |
| Disturbance Group | 0.5241 | 0.6002 |
| Measurement Method | -4.9240 | <0.0001 |
| Plant Group  × Disturbance Group | -0.6388 | 0.5230 |
| Plant Group  × Plant growth Form | 4.9757 | <0.0001 |
| Plant Group  × Measurement Method | -0.3023 | 0.7625 |
